# Retraction: HOTAIR contributes to cell proliferation and metastasis of cervical cancer via targeting *miR-23b*/MAPK1 axis

**DOI:** 10.1042/BSR-2017-1563_RET

**Published:** 2021-06-08

**Keywords:** HOTAIR, MAPK1, miR23b

This Retraction follows an Expression of Concern relating to this article previously published by Portland Press.

This article is being retracted from *Bioscience Reports* at the request of the Editor-in-Chief and the Editorial Board following receipt of a notification from a reader, alerting the Editorial Board to Figures 2C, 3C, 5E, 6J and 7A which contain overlaps in data and duplication of images with several other publications.

The authors have been contacted in regards to the retraction, and have not responded to the Journal's queries and the concerns raised. Given the extent of the issues raised, the Editorial Board stand by the decision to retract the article.

